# The diagnostic value of multichannel VEPs for children without nystagmus

**DOI:** 10.1007/s10633-025-10020-7

**Published:** 2025-04-17

**Authors:** Siân E. Handley, Joanne Cowe, Lisa Tucker, Oliver R. Marmoy, Dorothy A. Thompson

**Affiliations:** 1 Great Ormond Street Hospital for Children, NHS Foundation Trust, London, UK; 2 UCL Great Ormond Street Institute of Child Health, University College London, 30 Guildford Street, London, UK; 3 University Hospitals of Leicester NHS Trust, Leicester, UK

**Keywords:** Multichannel, Pattern reversal visual evoked potential, VEP, Children, Half field, Hemifield, Trans-occipital distribution

## Abstract

**Purpose:**

This study explored the clinical value of routine multichannel pattern reversal visual evoked potential (prVEP) recordings in children without nystagmus.

**Methods:**

A single centre, retrospective case note review was carried out of children without nystagmus who had multichannel prVEP recordings from midline, O1 and O2 electrodes referred to Fz to an ISCEV large check (50’ check width), reversing 3/s in a full 30° field and right and left 0–15° half fields, during 2020. Full-field (FF) prVEPs were classified as abnormal if midline P100 amplitude and peak time fell outside reference limits. Trans-occipital distribution asymmetry was defined as differences ≥ 20% amplitude between FF-prVEP the O_1_ and O_2_ at the peak time of the midline P100. Half field (HF) prVEPs acted as the gold standard discriminator of abnormality. The trans-occipital distribution and amplitude of the HF-prVEP ipsilateral positive peak (iP100) were compared for each eye.

**Results:**

FF-prVEP and HF-prVEP data from 63 children were classified. Group 1, 7/63 (11%), had abnormal midline FF-prVEP evidence of visual pathway dysfunction, whilst Group 2, 56/63 (89%), had normal midline FF-prVEPs. Group 2 was subdivided further according to the trans-occipital distribution of FF-prVEPs followed by HF-prVEPs. Group2A, 14/56 (25%), had symmetrical FF-prVEP distribution and normal HF-prVEPs. Group2B, 31/56 (55.4%), had asymmetrical FF-prVEP distribution, but lateralised HF-prVEPs that explained the FF-prVEP asymmetric distribution. Group2C, 11/56 (19.6%), had HF-prVEP evidence of pathway dysfunction with symmetric (n = 2) or asymmetric (n = 9) FF-prVEP distributions. Common referral reasons in all groups were reduced vision, glioma, craniopharyngioma, epilepsy presurgical evaluation, craniosynostosis, papilloedema/disc drusen, with various other specific conditions.

**Conclusions:**

Multichannel prVEPs add value to investigations of reduced or unexplained vision in children without nystagmus. Visual pathway abnormalities would not have been identified without a multichannel FF- or HF-prVEP in 11/56 (19.6%) of children in this study who had normal midline FF-prVEPs.

**Supplementary Information:**

The online version contains supplementary material available at 10.1007/s10633-025-10020-7.

## Introduction

Multichannel visual evoked potential (VEP) recordings have long been advocated in neuro-ophthalmology and paediatric practice [1–4]. Indeed, early versions of the ISCEV VEP Standard [5, 6], specified three active recording channels (O1,Oz,O2) for the simultaneous assessment of pre-chiasmal, chiasmal, and post-chiasmal function, but these Standards were cited rarely. The 2010 VEP Standard review committee attributed the lack of citations to the complexity of multichannel recording [7] and removed multichannel VEPs as an essential requirement from the following Standard revision [8].

The current (2016) and 2025 draft update ISCEV Standard for clinical VEPs [9, 10] defines protocols for a single midline channel to assess pre-chiasmal function whilst noting that extended multichannel protocols are needed to evaluate post-chiasmal lesions.

This change may be responsible for a disparity of VEP practice between new and longer established electrophysiology practices. In 2006 the ISCEV Committee for Pediatric Clinical Electrophysiology published guidelines and included the results of a 2000 survey of ISCEV members [11]. The majority (94%) of respondents indicated that they used a multichannel electrode configuration for paediatric VEPs which was specified in the 1996 VEP Standard current at the time [5]. Therefore, longer established clinics have likely incorporated multichannel VEP as routine. Laboratories set up more recently following the current VEP Standard [9] may only use a multichannel array when indicated, such as investigating chiasmal misrouting in children with nystagmus.

The choice of a single or multichannel VEP protocol may therefore depend upon the referral. The information provided in referrals for paediatric electrophysiology services can be vague such as ‘reduced vision’ or ‘unexplained visual loss’, reflecting the non-specific nature of signs and symptoms often encountered in children. In these cases, it is not clear if chiasmal or post-chiasmal function is a clinical concern or part of a differential diagnosis.

There are no published ISCEV extended guidelines for multichannel or hemifield VEP recording, at the time of writing, though the use of multichannel pattern reversal VEPs (prVEPs) is not new. Barrett et al. [12] were the first to describe the paradox of the half field prVEP trans-occipital distribution, which is associated with the monopolar electrode montage described in the ISCEV VEP standard. These methods have been used in several subsequent studies to determine chiasmal and retro-chiasmal field defects [1–4, 13, 14]. Mellow et al. [13] showed how hemianopic field defects can be masked in a single midline full-field prVEP (FF-prVEP) but revealed using a multichannel hemifield prVEP (HF-prVEP).

The aims of this study were to determine any benefit and clear clinical indications for the use of multichannel prVEPs in children without nystagmus, by comparing the classification of visual dysfunction based only upon the midline FF-prVEP to that based upon the trans-occipital distribution of the FF-prVEP and subsequent HF-prVEPs.

## Methods

### Study design

A retrospective case note review was conducted considering consecutive patients who attended a tertiary paediatric centre (Great Ormond Street Hospital for Children, London, UK) for visual electrophysiology testing during the year 2020. All children attending the centre as a minimum have routine skin flash ERGs alongside flash and pattern VEPs [14].

### Patient inclusion

Patient data were included if they had monocular multi-channel prVEP results for the ISCEV large check width (50’) produced from full field (30°) and 0–15° right and left hemifields. The HF-prVEPs were taken as the gold standard discriminator of hemifield dysfunction [13, 15]. Patients need reliable fixation to perform HF-prVEPs. Therefore, any children presenting with nystagmus were excluded. MRI findings were not included as these either were not yet performed or not available if performed external to the referral centre or had variable time intervals between scan and the VEP study date. Reports were checked for comments on electrode position and were excluded if these comments reported VEP data had been acquired with electrodes in an adapted or non-protocol montage (i.e. avoiding recent surgical sites or highly atypical skull anatomy).

### Data acquisition

All prVEPs were recorded using Diagnosys LLC, Espion E3 hardware and software version number V6.64.9 with amplifier input gain 8. A trans-occipital array (O1, midline, O2) of silver-silver chloride electrodes, referred to Fz, were acquired as per the laboratory protocol. Skin was prepared with abrasive gel (Nuprep®, Weaver and Company) and electrodes affixed using conductive paste (Elefix, Nihon Kohden) and secured with 3M™ Coban headband. A minimum of 30 trials were averaged and repeated at least twice to ensure repeatability. A virtual channel displayed a subtraction of prVEPs on the lateral channels, O1-O2 difference_,_ (i.e. left -right occiput), in real time. This helps to identify asymmetries of trans-occipital VEP distribution whilst the patient is being tested. When asymmetries are seen at the start of data acquisition electrode position is re-checked. Comments on electrode position, checking and repositioning (if required) are documented in the final report. Impedances remained equal and below 5kΩ. A filter bandpass of 0.3-100Hz was used with an acquisition time window of 15ms pre stimulus and 285ms post stimulus. The sample frequency was 1kHz, with a pattern reversal rate of 3.15rps of 50’ check widths presented on a plasma display panel of mean luminance 82 cd/m^2^, providing a 30° field at a 1m viewing distance. For half field stimulation the right or left half of the screen was masked with a uniform, grey background of equal mean luminance to the checkerboard stimulus presented on the other side. Fixation was monitored throughout using a video camera. Data collection was paused when fixation wavered. Good fixation was encouraged by a second vision scientist, situated next to the display but out of the stimulated field. A DVD of the patient’s choice was visible on the plasma display between periods of checkerboard stimulation and the soundtrack was audible continuously to encourage compliance.

###  Nomenclature of full field and hemifield prVEP components

The multichannel montage of occipital electrodes (O1 and O2) referred to Fz combined with a large, 30 degree stimulus field and 50’ check widths provides a well characterised trans-occipital distribution of FF-prVEPs and HF-prVEP components [1, 12, 16]. The FF-prVEP is dominated by the P100 which typically is largest at the midline and distributed symmetrically about the midline with lower positive peak amplitudes over O1 and O2. Half field (0–15 degree) stimulation localises the generating activity to one hemisphere. In describing HF-prVEPs Barrett et al. [12] used the terms macular and paramacular components, and paradoxical lateralisation. Paradoxical lateralisation is named because with the conventional electrode montage (O1 and O2 referred to Fz), the P100 will lateralise over the same occiput as the half field stimulated, instead of being detected directly over the generating hemisphere. The HF-prVEPs components that lateralise over the ipsilateral occiput to the stimulated field are considered macular components, with the prefix “i” for ipsilateral, named iN75-iP100-iN145 (such as produced on O2, the right occiput, by RHF stimulation of the left hemisphere) [12, 14]. Over the contralateral occiput to the stimulated half field a negative component is seen at a similar time (cN105) which is considered to be of paramacular origin, with the prefix “c” for contralateral. The cN105 can be preceded and followed by a small positivity producing a positive–negative- positive complex (or cP-N-P).

### Data analysis

The patients were first classified as having normal or abnormal FF-prVEP by comparing P100 measured at the midline electrode to laboratory reference ranges for amplitude and peak time [17]. Group 1 were patients with abnormal FF prVEPs at Oz in one or both eyes i.e. visual loss was identified for these patients using a single midline recording channel. Group 2 patients had normal midline FF-prVEP to 50’. Group 2 patients had no vision loss identified using the midline channel ISCEV Standard VEP protocol [9, 10].

Group 2 patients with normal midline FF-prVEP P100 were further classified into three subgroups based upon the trans-occipital distribution of the FF-prVEP over the three active channels, and then by the HF-prVEPs, (Group 1 were excluded from this analysis).

Symmetrical and asymmetrical trans-occipital distributions of FF-prVEPs were defined by the percentage difference in amplitudes between O1 and O2 measured at the same peak time as the midline P100. As described by Mellow et al. [13], the FF-prVEP trans-occipital distribution was considered symmetrical when the difference in lateral channel amplitude was 20% or less at the time of P100 at the midline, and 10ms or less difference in maximal positive peak times on O1 or O2. Larger differences in amplitude and/or peak time were considered an asymmetrical trans-occipital distribution.

**Fig. 1:**
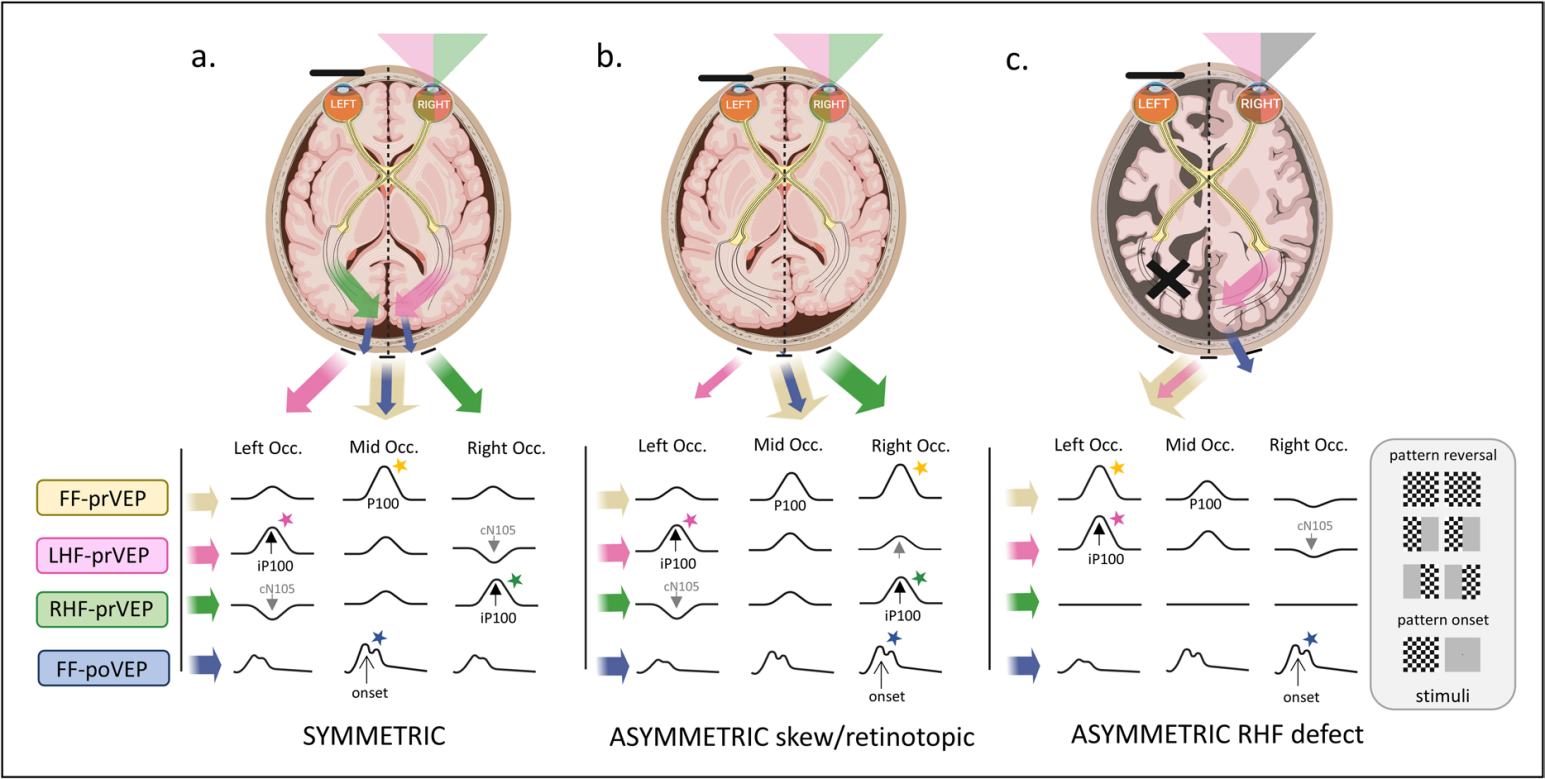
Schematic visualization of the trans-occipital distribution of pattern VEPs. Created in BioRender. Handley, S. (2025) https://BioRender.com/r49b873. Three scenarios are described. **a** Symmetric distribution. The brain figure shows two hemispheres symmetrically positioned about a midline axis (dotted line). When the full, 30 degree field, of the RE is stimulated, each hemisphere and each central 0–15 degree HF contribute equally to the FF-prVEP. The summation of responses from the right and left hemisphere is maximal at the midline (Oz). The laterally positioned electrodes show smaller, but similar positive peaks over the right and left occiputs (top row, following the yellow arrow). The poVEP is also maximal at the midline (bottom row, blue arrow). If the RHF or LHF is independently stimulated the response of one hemisphere is unopposed. The resulting HF-prVEP is detected optimally over the opposite hemisphere, ipsilateral to the stimulated field, (following green RHF or pink LHF arrows). This is called paradoxical lateralization (11). For example, LHF stimulation of the right hemisphere produces an iP100 on the left occiput, (pink arrow and star). The opposite occurs for the RHF. The RHF-prVEP produces an iP100 which is maximal on the right occiput (green arrow and star). On the contra-lateral occiput there may be a smaller positive peak or cN105. The cN105 represents paramacular components and the iP100 macular components. **b** Asymmetric, retinotopic distribution. Frequently, the two hemispheres are not completely symmetrical about a midline axis. One hemisphere, often the left, curves around the right hemisphere. This is termed an occipital petalia. This is seen in Fig. 1b as an extension of the left occipital lobe over the dotted axial midline. The occipital petalia will change the projection angle of the VEP generators and can produce an asymmetric occipital distribution of FF-prVEP and FF-poVEPs. As seen in this schematic the response summation of both hemispheres has a similar maxima for FF-pr-VEP and FF-poVEP, both lateralize over the same occiput. The HF-prVEPs can confirm if both central half fields are responsive in such circumstances. For example, here the FF-prVEP is smaller over the left occiput suggesting the contribution of the LHF maybe reduced. Independent stimulation of each half field produces an iP100 from the LHF as expected over the left occiput and confirms the LHF is responsive. On the other occiput the LE LHF cN105 is now a small positive peak. The RHF iP100 is lateralized as expected and is maximal over the right occiput. **c** Half field defect. In this example the hemispheres are symmetrical about the midline axis (dotted line), but the left hemisphere is malformed and dysfunctional. It doesn’t contribute to the FF-prVEP. The FF-prVEP therefore resembles the waveform and trans-occipital distribution of the functioning LHF (pink arrow and star). The FF-prVEP is maximal over the left occiput. The onset VEP generators faithfully follow the stimulated hemisphere, in this case the right hemisphere and the FF-poVEP is maximal over the right occiput, the opposite side of the FF-prVEP. This different lateralization of the major positive peaks of FF pr-VEP and FF-poVEP suggests true HF loss. This comparison is helpful in paediatric practice when children are unable to comply with the fixation needed for HF stimulation

HF-prVEP ipsilateral and contralateral waveforms from HF of each eye are expected to be similar on O1 and O2. The HF-prVEP morphology from each HF of each eye were examined individually and then overlaid for comparison. The summation of RHF and LHF pr-VEP waveforms on O1, Oz and O2 were compared with the FF-prVEP waveforms at each electrode for each eye. In addition, ip100 amplitude and peak time for each eye and HF were collated. Based on the measurements and visual inspection, HF-prVEPs were described as normal or abnormal. In combination with FF-prVEPs this gave 3 subgroups which are illustrated in Fig. 1. Group 2A: Symmetrical FF-prVEP distribution with normal HF-prVEPs, (Fig. 1a). Group 2B: Asymmetric FF-prVEP distribution with normal HF-prVEPs that when summed explain the skewed FF-prVEP trans-occipital distribution. Termed a retinotopic distribution as likely reflects individual variation in VEP generator orientation or cortical architecture, such as an occipital petalia, (Fig. 1b). Group 2C: HF-visual dysfunction evidenced by an abnormal HF-prVEP iP100 in presence of either symmetrical or asymmetrically distributed FF-prVEP, (Fig. 1c).

## Results

Within the sampling period of the year 2020, 63 patients met the inclusion criteria. Data were captured during the COVID-19 pandemic when fewer referrals were received than is typical. Patient ages ranged from 4.4 – 16.9 years (y), with a median of 10.5y. The grouped patient results are summarised in Table 1. The measurements and distribution of FF-prVEP and HF-prVEP for individual patients in Group 2 are detailed in the Supplementary Table 1.

**Table 1:** Summary result table of findings in each group

Group		N (%)	Age y median (range)	Clinical referral reasons	FF-prVEP distribution
1	Group 1 identified as ABNORMAL FF-prVEP on midline – one or both eyes				
	Abnormal Oz FF-prVEP	7 (11%)	11.8 (7.9–15.2)	reduced vision (3/7), glioma (2/7), craniopharyngioma (2/7)	Abnormal FF-prVEP at midline for one or both eyes
2	Group 2 identified as NORMAL FF-prVEP on midline which are then sub classified by HF-prVEP findings				
	Normal Oz FF-prVEP	56 (89%)	10.5 (4.4–16.9)	varied reasons; see subgroups below	Normal FF-prVEP at midline with P100 within reference range
Sub-groups of Group 2 based on trans-occipital distribution of FF-pr-VEP and HF-prVEP findings (described in Fig. 1.)					
2A	Group 2A: No visual field dysfunction AND symmetrical FF-prVEP distribution, normal HF-prVEPs				
	Symmetrical FF-prVEP Normal HF-prVEP iP100	14 (22.2%)	12.3 (6.6–16.2)	reduced vision (3/14) craniosynostosis (4/14) papilloedema / drusen (3/14) IIH (2/14) other specific issues (2/14)	Minor FF-prVEP asymmetry ≤ 18% Range 0% to 18% (median 9% IQR 10) Inter ocular % difference of trans-occipital asymmetry range 0% to 12% (median 8% IQR 6)
2B	Group 2B: No visual field dysfunction BUT asymmetrical FF-prVEP distribution, normal HF-prVEPs				
	Retinotopic distribution HF-prVEP iP100 evident	31 (49.2%)	9.4 (4.4–16.9)	reduced vision (10/31) epilepsy pre surgical evaluation (3/31) craniosynostosis (7/31) glioma (2/31), IIH/papilledema/drusen (4/31) other specific issues (5/31)	Asymmetrical but normal retinotopic distribution; FF-prVEP trans-occipital asymmetry up to 95% Range 8% to 95% (median 39% IQR 29) Inter ocular % difference of trans-occipital asymmetry range 1% to 49% (median 9% IQR 14)
2C	Group 2C: Visual field dysfunction FROM HF-deficit, asymmetric FF-prVEP or symmetric FF-prVEP with abnormal HF-prVEPs				
	Abnormal HF-prVEPs, One eye 8 patients Both eyes 3 patients	11 (17.5%)	9.8 (5.1–16.3)	reduced vision (7/11), glioma (2/11), epilepsy pre-surgical evaluation (1/11) optic atrophy (1/11)	Symmetrical FF-prVEP (2/11) Asymmetrical FF-prVEP (9/11), FF-prVEP trans-occipital asymmetry up to 70% Inter ocular % difference of trans-occipital asymmetry range 0% to 47% (median 12% IQR 15.5)

The numbers of patients in each Group 1 and 2, and subgroups A,B and C are described with their age, range of referral reasons and summary of the range FF-prVEP trans-occipital asymmetry and the interocular difference in asymmetry detailed. The full findings for all patient in Group 2 are given in Supplementary Table 1

### Group 1 abnormal midline (Oz) full field pattern reversal VEPs

Seven children (11.1% of total sample) had abnormal FF-prVEPs at the midline from one or both eyes.

### Group 2 normal midline (Oz) full field pattern reversal VEPs

The midline FF-prVEPs of the remaining fifty six children (89% of total sample) had P100 peak time and amplitude within laboratory reference range. Multichannel FF and HF-prVEPs showed no evidence of visual dysfunction in 45/56, comprising Group 2A and 2B, but revealed visual dysfunction in 11/56 patients (19.6% of group 2, 17.5% of total sample) in Group 2C.

In Group 2B the asymmetrical FF-prVEP was homonymously distributed i.e. smaller over the same occiput for both eyes in all 31 patients (Supplementary Table 1). Mostly the FF-prVEP was smaller over the left occiput for both eyes (in 22, 71%) and smaller over the right occiput for both eyes in 9 (29%).

**Fig. 2:**
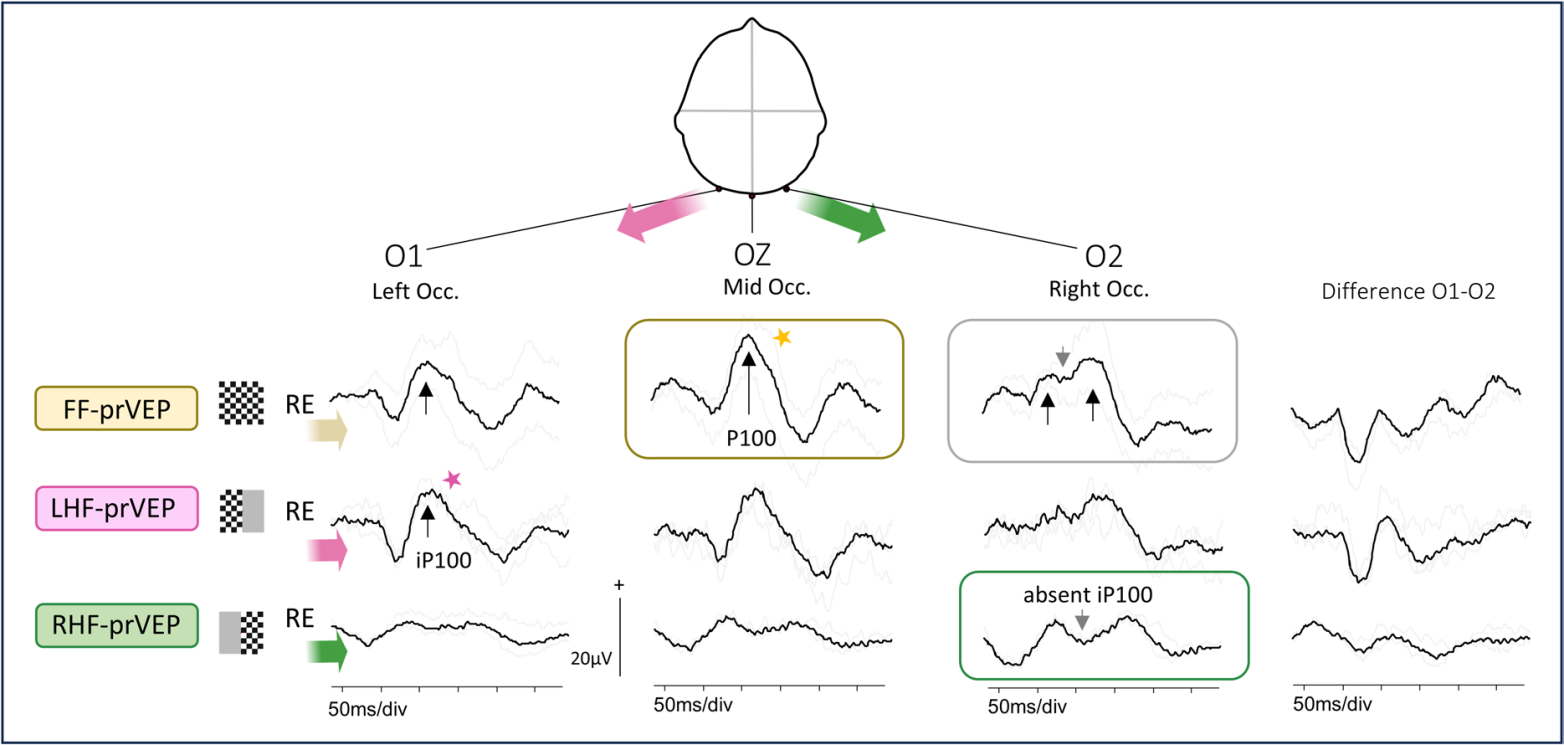
Normal FF-prVEP at Oz. This is an example of a ‘normal’ RE FF-prVEP from a patient in Group A. The LE FF-prVEP P100 peak time was prolonged at 133 ms, thus dysfunction was detected by the ISCEV Standard FF-prVEP at Oz, but not localized. The RE P100 at Oz has amplitude and peak time within reference limits, but the waveforms on the lateral channels O1 and 02 are dissimilar. The FF-prVEP at right occiput, O2 comprises 2 positive peaks (arrowed in the grey box) compared to a single peak on O1. HF-prVEPs show the iP100 from the RE RHF-prVEP is absent, which leaves a bifid waveform on O2. This indicates a RHF field defect (green box). The LHF-prVEP iP100 on O1 is well defined and shows the RE LHF is functional (pink star). The FF-prVEP trans-occipital distribution resembles that of the functioning LHF-prVEP. This 9y patient had a craniopharyngioma and is a clinical example of the trans-occipital distribution shown in Fig. 1c

**Fig. 3:**
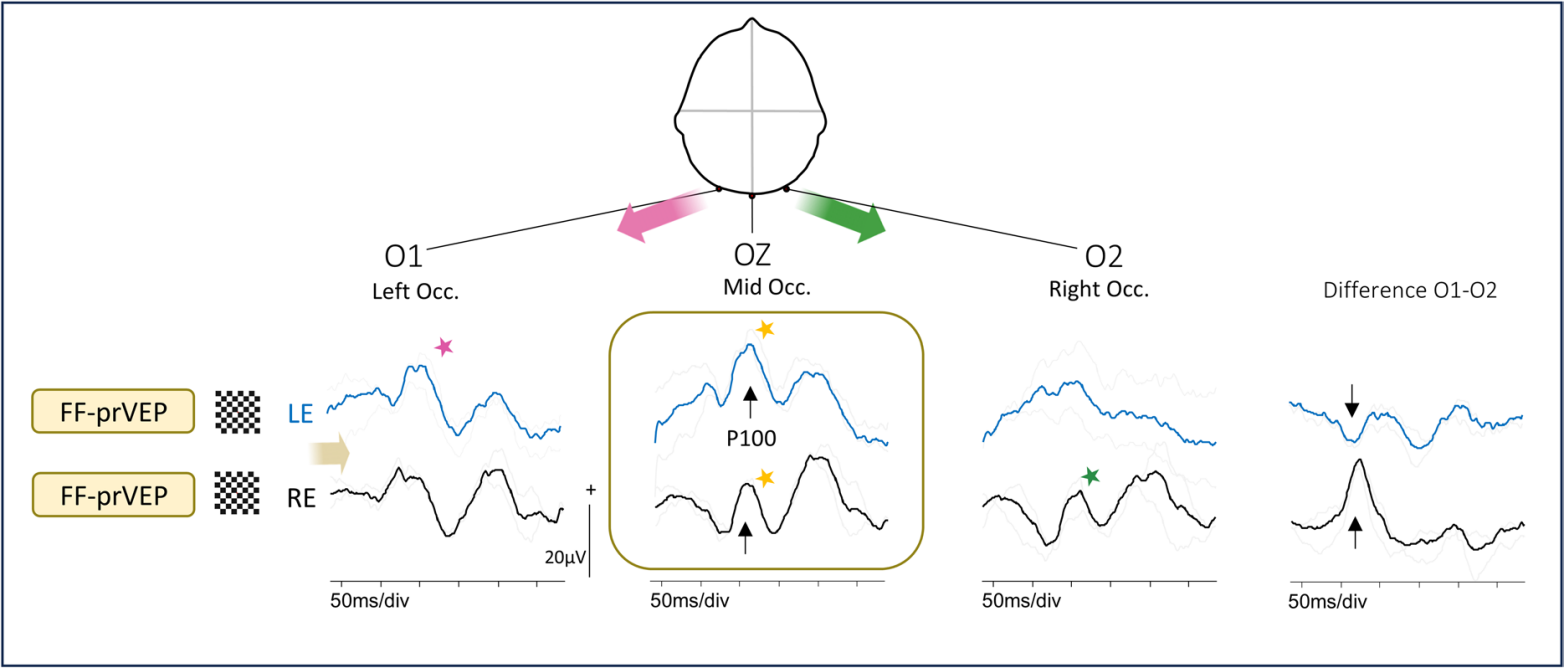
Crossed asymmetry. The midline FF-prVEP P100 from each eye is within reference range. In isolation this might suggest normal macular pathway function from each eye, (box with yellow stars marking the peaks) BUT there is an inter-ocular difference of waveform morphology on the lateral channels. This is visualized clearly when a subtraction of O1-O2 is taken, displayed in the virtual difference channel. This shows a polarity reversal (arrowed), a crossed asymmetry, which is a sign of chiasmal dysfunction or disproportion. This is a clinical case of a 6y child referred with unexplained bilaterally reduced vision LogMAR RE 0.60 L 0.34 with RE esotropia. Patient 15

**Fig. 4:**
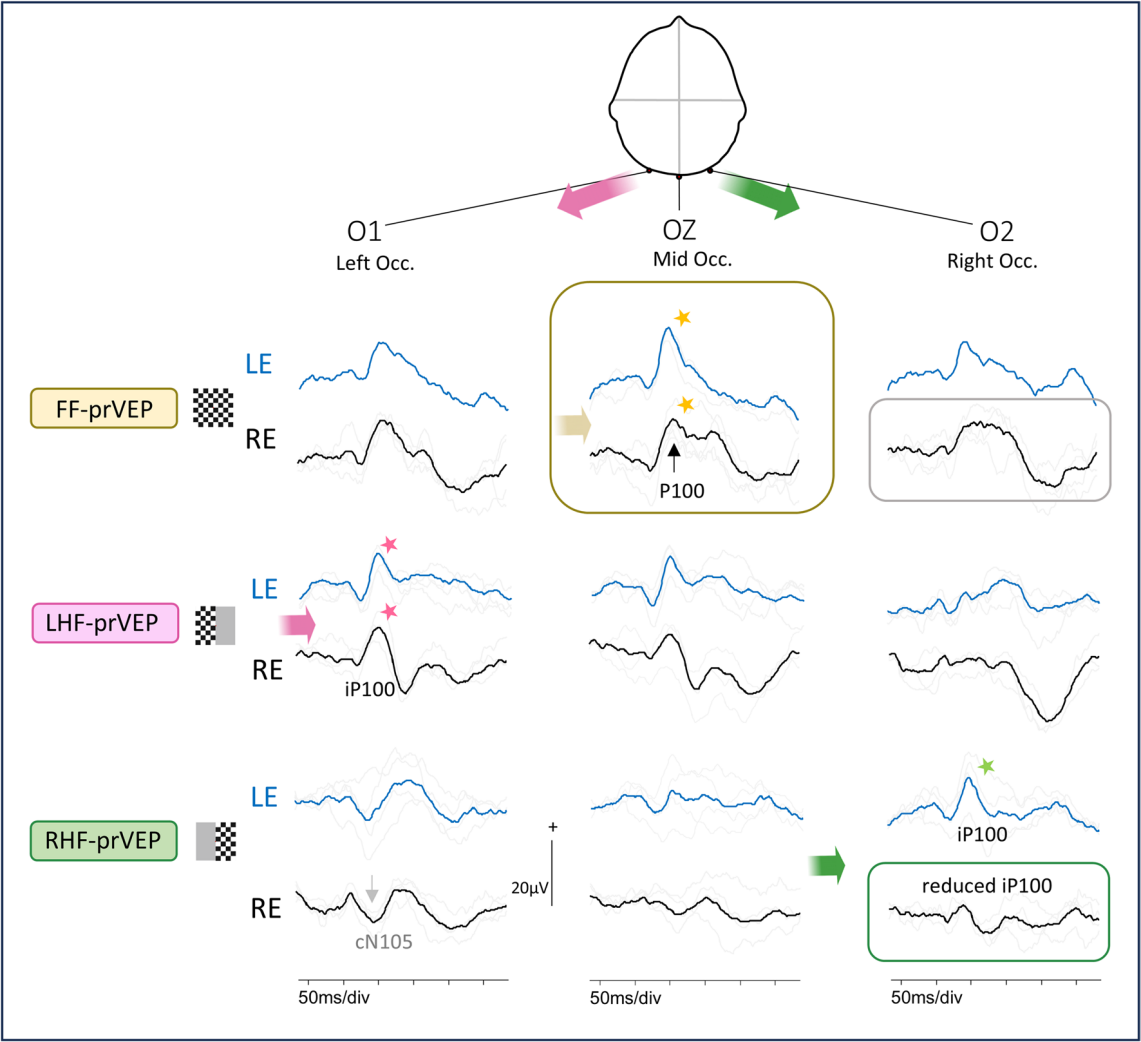
Inter-ocular difference FF-prVEP waveforms at O2, right occiput. The RE FF-prVEP positive peak is broad and later peaks, which typically arise from paramacular regions, are prominent. This is suspicious of RE RHF dysfunction confirmed by HF-prVEPs that show a reduced iP100 from the RE RHF. Patient 16

**Fig. 5:**
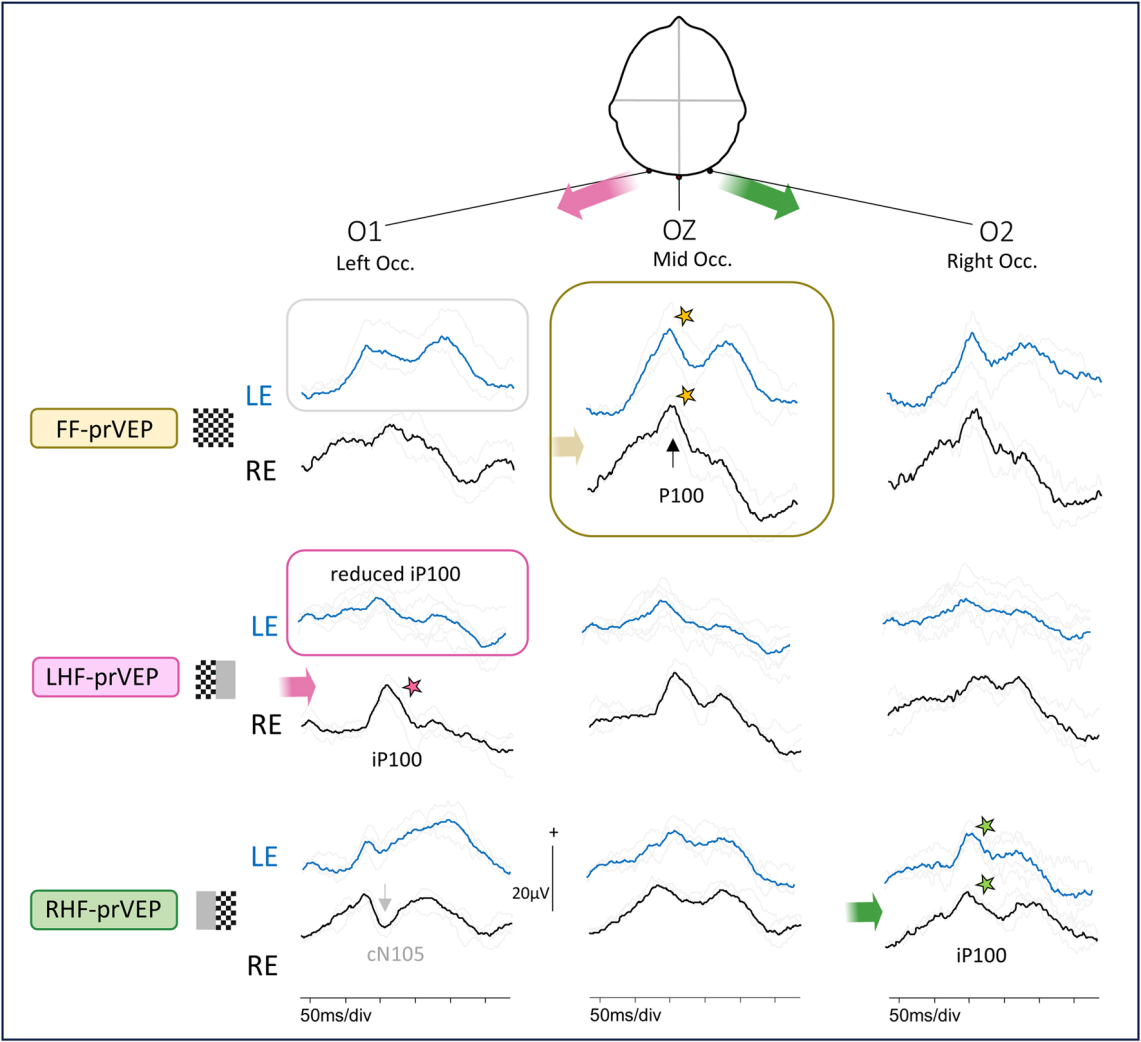
LE FF-prVEP atypical waveform at O1, left occiput. The midline P100s from each eye are similar when overlaid and amplitude and peak time are within reference range – without lateral channel this might suggest normal macular pathway function from each eye BUT there is an inter-ocular difference of FF-prVEP waveforms on O1. The LE FF-prVEP at O1 has a bifid morphology with an atypically early P-N-P complex (grey box). This suggests pronounced paramacular contribution to the LE LHF. This is confirmed by HF-prVEPs that show a reduced iP100 macular component from the LE LHF, (pink box), revealing the paramacular waveform. Patient 17

**Fig. 6:**
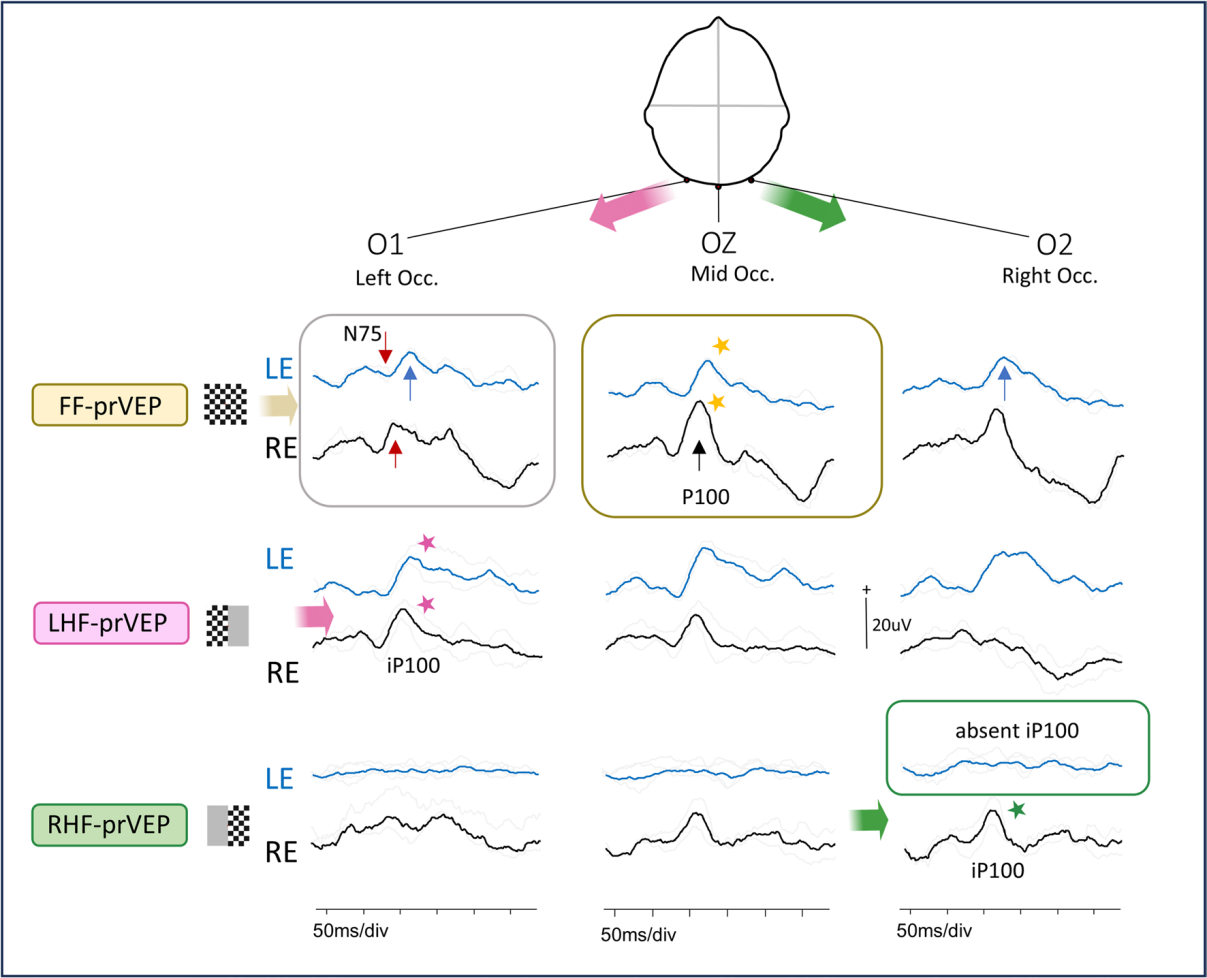
Symmetrical LE FF-prVEP distribution with RHF iP100 loss. The LE FF-prVEP P100 is symmetrically distributed with similar waveforms on O1 and O2, (positive peak blue arrows). The LE midline P100 peak time is within reference range, but slightly later than the RE P100. The RE FF-prVEP is asymmetrically distributed. This is seen at O1 where LE and RE FF-prVEPs are dissimilar with an apparent polarity reversal warning of some dysfunction. The LE RHF-prVEPs shows this is associated with an absence of a response from the LE RHF, (green box). Patient 14

**Fig. 7:**
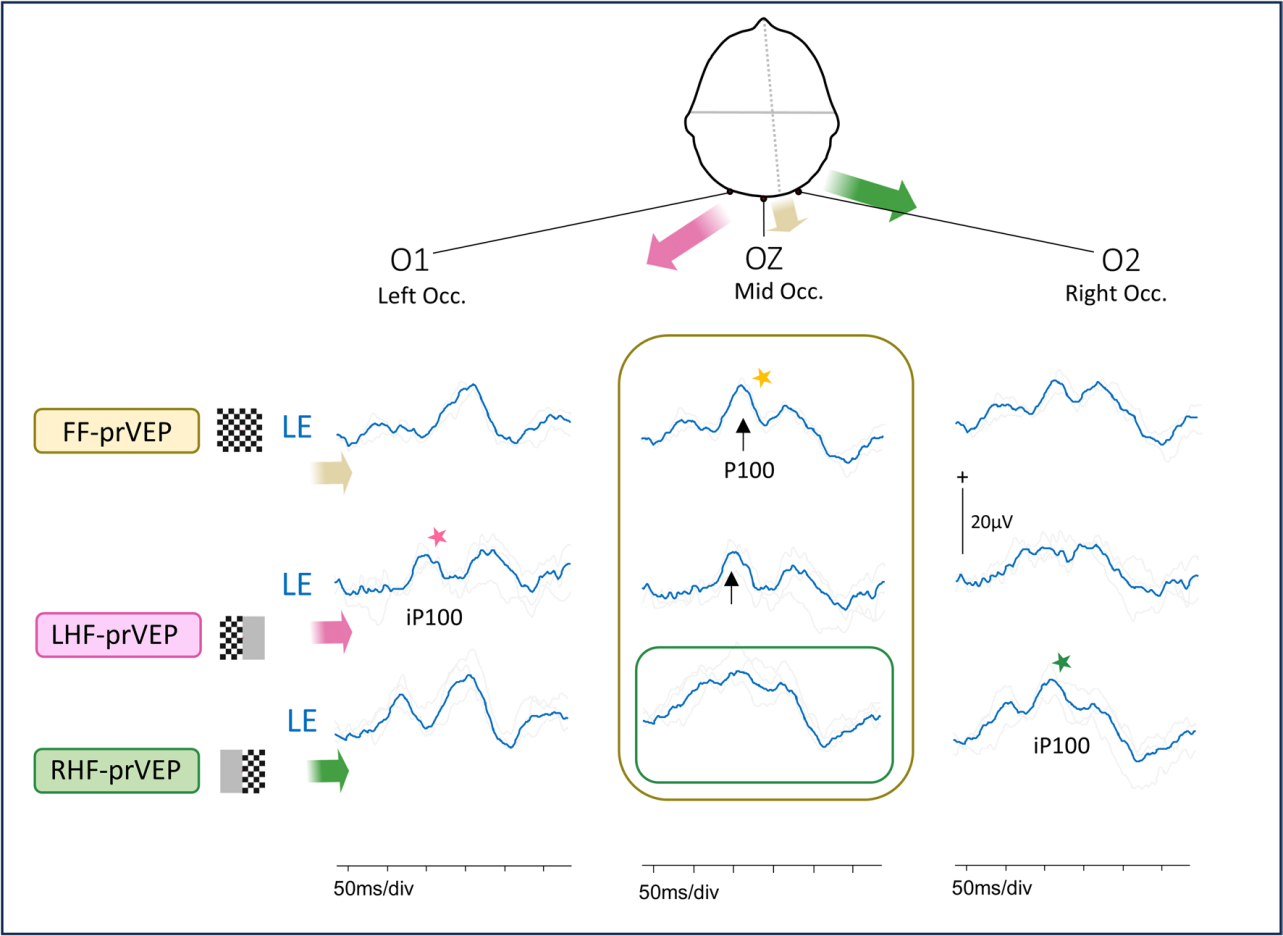
Lateral channels are needed to evaluate HF-prVEPs. Half field pr-VEP positive peaks are seen on the midline channel in many subjects, but in some there is a difference in the orientation of a retinotopic generator, as illustrated in the schema Fig. 1b. In this case example an inspection of the HF-prVEPs at the midline suggests a deficit of the RHF (green box), because the waveform at Oz is ill-defined and small amplitude. On the right occiput however the lateralized iP100 is clearly seen (green star). This shows the RHF is functional. Without the lateral channels the HF-prVEP may have been interpreted incorrectly as a LE RHF defect. Patient 22. In Fig. 2 the RHF-prVEP was similarly ill-defined on Oz, but in contrast the iP100 is absent confirming a RHF defect in this patient

Figures 2, 3, 4, 5, 6, 7 provide clinical examples of FF-prVEP with abnormal HF-prVEPs (Figs. 2, 3, 4, 5 and 6 from patients in Group 1 and 2C) and normal, but lateralised HF-prVEP iP100 (Fig. 7 Group 2B).

## Discussion

Of the sixty three cases reviewed in this study, eighteen (28%) patients had visual pathway dysfunction, but only 7/18 (39%) patients had dysfunction identified by a single, midline channel prVEP. The others 11/18 (61%), constituting 17.5% of the total patients sampled, would have been incorrectly reported as having ‘normal visual pathway function’ without the use of multichannel prVEPs. Additionally, although a single channel VEP may identify dysfunction, it doesn’t localise the site of abnormality within the visual pathway.

This study cautions that a midline FF-prVEP within reference range does not exclude visual pathway dysfunction. In this series of 56 young patients with normal midline FF-prVEP eleven (19.6%) had abnormal multichannel FF- or HF-prVEPs. Diagnosis of visual pathway dysfunction would have been missed or delayed in these children if only a single midline prVEP had been recorded. Some of these children were referred for investigation of reduced vision (20/56 patients), which may not necessarily prompt a specific multichannel VEP investigation of chiasmal or post-chiasmal dysfunction.

A normal midline FF-prVEP comprises contribution from both hemispheres as illustrated in Fig. 1a. A robust FF-prVEP from just one hemisphere can produce a midline P100 with amplitude and peak time within the laboratory’s reference range and therefore mask a hemisphere, chiasmal or monocular hemifield dysfunction, as illustrated in Fig. 1c with a clinical example Fig. 2. Adding together the left and right HF-prVEP waveforms at O1, Oz and O2 approximates the FF-prVEP waveform at O1, Oz and O2 and explains the FF-prVEP trans-occipital distribution for that eye [16]. Examining the HF-prVEP waveforms in this way can help explain if asymmetries of FF-prVEP trans-occipital distribution are likely due to pathology, an absent HF-prVEP iP100 (Fig. 1c), or individual cortical architecture (Fig. 1b), or technical reasons such as asymmetric or misplaced electrode positioning [13, 15].

Asymmetrical FF-prVEPs explained by HF-prVEPs (Group 2B) were more likely to be smaller over the left occiput than the right (ratio 1:2.5 R:L n = 31), similar to the findings reported by Mellow et al. (1:5 R:L homonymous retinotopic data n = 13) [13]. They also reported cautionary cases of normal, symmetrical FF-prVEPs, when only HF-prVEPs unmasked a defect [13]. There were two clear examples of this in our data set, (patient 10 RE, and patient 14 LE (shown in Fig. 6), where the FF-prVEPs were symmetrically distributed, but the entire contribution came from the remaining hemifield which was symmetrically distributed (mechanism illustrated in Schematic Fig. 1c). Both examples in our series had optic pathway tumours and were being monitored for progression. Missing this hemifield deficit could potentially impact their diagnosis, monitoring and care.

The extent of the trans-occipital asymmetry does not differentiate between a trans-occipital asymmetry raising concern (i.e. a half field defect Group 2C) or associated with individual anatomy (i.e. retinotopic distribution Group 2B). Larger trans-occipital asymmetries of up to 95% were seen in the retinotopic Group 2B compared to up to 70% in the Group 2C with half field deficits. Indeed 19/62 eyes (30.6%) had a retinotopic asymmetry of > 50% in this study which is similar to the 14/38 (37%) of eyes reported by Mellow et al. [13]. Together these data suggest approximately a third of asymmetric FF-prVEP occipital distributions, due to individuality retinotopic mapping and orientation of cortical generators, are larger than 50%.

In this study HF-prVEPs are used as a gold standard to define pathway dysfunction. Although the patients’ ages ranged from 4.4 – 16.9 years at testing and show that HF-prVEP tests can be achieved from quite a young age, the steady fixation required is not always feasible for children. For patients who cannot perform HF-prVEP it is possible to compare paradoxical FF-prVEP trans-occipital asymmetries with the faithful distribution of full field pattern onset VEPs over the activated hemisphere to distinguish pathology [15, 18], as visualised in Fig. 1 a,b and c. Lateralisation of P100 and the main FF-poVEP positive peak to opposite hemispheres signposts the need for further investigation. Although reasonable to assume the specificity and sensitivity of FF-poVEP in isolation, or in combination with FF-prVEP, to detect pathology at the chiasm at least, may be similar to that reported for the detection of chiasmal misrouting in albinism, as yet this has not been established.

Prompt identification of chiasmal and post-chiasmal dysfunction is important as delayed assessment and/or misdiagnosis may result in severe and irreversible visual loss. Furthermore, even anterior visual pathway dysfunction may sometimes affect one hemifield more than another [19]. Visual electrophysiology referrals may not state whether chiasmal or retrochiasmal dysfunction is suspected. Furthermore, in paediatric practice children may not complain of typical symptoms of vision loss or field defect [20, 21]. Further investigations such as neuroimaging may not have been requested for children with non-specific ‘reduced vision’. Whilst formal perimetry is possible in typically developing children from the age of 5 years [22] an electrophysiological abnormality can precede the perimetric visual-field defect in some cases and can be achievable before formal perimetry in children [15]. Of note, the referrals for four children (Supplementary Table 1 patients 8, 26, 35 and 56) documented specifically that they were unable to perform reliable visual field tests, but all children managed reliable multichannel HF-prVEP tests. When planning a test strategy there is evidence in the literature that multichannel electrode arrays combined with FF-prVEP and HF-prVEP significantly improves the sensitivity of the VEP in detecting post retinal visual field defects (for a review see Handley et al. [15]).

Whilst important to understand the referral reasons for visual electrophysiology investigation when choosing and optimising the test protocols for a lab and/or the test strategy for individual patients, there was no clear distinction between the referral indications for children in Group 1 identified with a single channel midline FF-prVEP compared to Group 2 identified by multichannel FF- and HF- prVEP recordings (Table 1). It is clear from the referral reasons for patients in Group 2 that a broad range of clinical queries benefited from the multichannel VEP recording. Some clinical referrals or conditions mandate a multichannel recording such as nystagmus and space occupying lesions. The multichannel VEP in children with glioma is as clinically important to exclude as well as identify visual pathway dysfunction because evidence of pathway dysfunction can a trigger the decision to treat. Of the 6 patients referred with glioma 4/6 were identified as having visual pathway dysfunction, 2 of whom only had abnormalities identified with multichannel VEP recording, their midline FF-prVEPs were normal (Supplementary Table 1 patients 10 and 11) and in one of these a symmetrical FF-prVEP distribution hid the hemifield deficit (patient 10).

Two patients were referred with abnormal foveal reflexes without nystagmus. These were later found to be associated with foveal hypoplasia and retinoschisis on OCT. Foveal hypoplasia is an indication for a multichannel VEP recording as it is associated with a number of conditions [23] including albinism, aniridia, prematurity, optic nerve hypoplasia, achromatopsia, cone-rod dystrophy, FHONDA (foveal hypoplasia, optic-nerve-decussation defects/dysgenesis) [24–26]. Therefore, supporting evidence such as the presence or absence of VEP misrouting may help in the differential diagnosis [4, 24–28]. Chiasmal misrouting typically is detected using mc poVEPs and/or mc flash VEP for younger children. The patient in this series with foveal hypoplasia, but no nystagmus, had a crossed asymmetry in keeping with that described in albinism identified in FF-prVEPs using the trans-occipital array. This would not have been identified using a single channel recording. Although potentially under-diagnosed, albinism without nystagmus is estimated to occur in 6–11% of people with albinism, meaning multichannel recording may not be selected based purely on the referral details [29, 30]. Of note children with nystagmus were excluded from this study and our findings highlight the added value of routine application of multichannel VEPs in particular for children referred with ‘reduced vision’. This was the most common referral question in this case series, given in 26/63 (41%) cases. Three patients in Group 1 were referred with reduced vision and had visual pathway dysfunction identified via a single channel midline FF-prVEP, but a further 6 patients presenting with reduced vision (patients 9,14–18 Group 2C) were only identified by using the multichannel FF- and HF-prVEPs (as illustrated in figs. 3,4,5 and 6). Therefore, it is not sufficient to perform single-channel VEPs for these patients as there is a potential to miss the diagnosis of visual pathway dysfunction in a substantial proportion of these cases.

The use of the referral question as a proxy or working clinical diagnosis is a limitation of this study as we do not have a definitive clinical diagnosis to calculate sensitivity and specificity of mcVEPs for each condition. Also our data reflect paediatric practice and the presentation in adult practice may differ. The placement of additional electrodes however is minimally time consuming and well accepted by children. A dialogue with referring clinicians is important to encourage the fullest clinical referral details but it is also likely that these details may be unknown or unavailable at the time of referral. The findings of this work therefore advocate that multichannel VEPs are beneficial in routine electrophysiology testing in children.

## Conclusion

This study of consecutive patients presenting without nystagmus highlights the diagnostic benefit of using multichannel prVEPs and supports its routine use for paediatric visual electrodiagnosis. Multichannel FF- and HF-prVEPs identified visual pathway dysfunction in 11/56 (~ 20%) children, who would have been misdiagnosed as having normal visual pathway function from the single midline FF-prVEP.

## Supplementary Information

Below is the link to the electronic supplementary material.

Supplementary file1 (PDF 110 KB)

## Declarations

### Statement on the welfare of animals

This research did not involve animals.
