# Supplementary material for: The diagnostic value of multichannel VEPs for children without nystagmus
Source: Doc Ophthalmol. 2025 Apr 17;151(1):31–44. doi: 10.1007/s10633-025-10020-7 (PMC12334463; doi:10.1007/s10633-025-10020-7)
Supplement: Supplementary file 1 — Supplementary file1 (PDF 110 KB) [file 10633_2025_10020_MOESM1_ESM.pdf]

TABLE S1. Patient group data. Groups A and B have normal HF-prVEPs. Group C have HF-prVEP defects. The group classification is described in Figure 1, schema of symmetrical (A), asymmetrical (B) FF-prVEPs and HF-prVEP (C).

ID	Age [years]	Ophthalmology referral	FF-prVEP trans-occipital asymmetry [%]		HF-prVEP iP100 amplitudes [µV]				HF-prVEP distribution	
			RE	LE	RE		LE		RE	LE
					RHF	LHF	RHF	LHF	RHF LHF	RHF LHF
**A.**		Normal - Symmetric FF-prVEP (normal FF-PrVEP at Oz and normal HF-prVEPs) n=14								
	symbols: ●= hemifield deficit, ○ = normal distribution of hemifields, ⊙ = atypical distribution of hemifields, ☼ = symmetric distribution of hemifields									
55	8.3	Craniosynostosis - sagittal	14% smaller L.Occ	17% smaller L.Occ	20	27	26	17	○○	○○
56	15.2	Craniosynostosis - sagittal	9% smaller R.Occ	0% symmetrical	8	11	10	8	○○	○○
60	11.9	Craniosynostosis - sagittal	9% smaller L.Occ	4% smaller L.Occ	22	21	20	16	○○	○○
54	8.7	Craniosynostosis - multisutural	9% smaller L.Occ	0% (symmetrical)	12	23	13	10	○○	○○
61	16.2	IIH	0% symmetrical	7% smaller L.Occ	13	7	15	11	○○	○○
63	12.3	IIH	0% symmetrical	8% smaller R.Occ	9	8	10	5	○○	○○
53	13.6	Pseudopapilledema<br>Elhers Danlos syndrome.	17% smaller L.Occ	14% smaller L.Occ	7	5	8	7	○○	○○
50	10.6	Optic disc drusen or<br>Papilledema	8% smaller R.Occ	18% smaller R.Occ	13	10	13	12	○○	○○
51	9.9	Optic disc drusen.<br>Alt XT mild amblyopia	14% smaller L.Occ	10% smaller L.Occ	9	15	12	12	○○	○○
52	15.1	Diplopia. Dizziness.<br>Non-specific symptoms	16% smaller R.Occ	6% smaller R.Occ	11	14	10	9	○○	○○
57	12.3	Peripheral field loss.<br>Unreliable visual fields.	14% smaller R.Occ	6% smaller R.Occ	18	24	16	14	○○	○○
58	15.6	Reduced vision.<br>Colour vision changes. Diplopia	7% smaller L.Occ	10% smaller L.Occ	12	9	14	13	○○	○○
59	16	Reduced vision.	0% symmetrical	0% symmetrical	9	11	8	10	○○	○○
62	6.6	Reduced vision.	16% smaller R.Occ	4% smaller L.Occ	24	27	26	31	○○	○○
**B.**		Normal - Asymmetric FF-prVEP Retinotopic (normal FF-prVEP at Oz and normal HF-prVEP) n=31								
	symbols: ●= hemifield deficit, ○ = normal distribution of hemifields, ⊙ = atypical distribution of hemifields, ☼ = symmetric distribution of hemifields									
19	10.8	Epilepsy presurgical evaluation<br>Left MCA stroke.	**38% smaller R.Occ**	11% smaller R.Occ	9	10	12	10	☼○	☼○
23	11.7	Epilepsy presurgical evaluation	**33% smaller L.Occ**	**43% smaller L.Occ**	12	16	12	12	○☼	○☼
42	15.8	Epilepsy presurgical evaluation<br>Focal epilepsy – temporal lobe	**38% smaller R.Occ**	**29% smaller R.Occ**	11	13	12	14	☼○	☼○
20	5.7	Craniosynostosis - sagittal	18% smaller L.Occ	**32% smaller L.Occ**	16	24	22	18	○☼	○☼
41	9	Craniosynostosis - sagittal	**35% smaller L.Occ**	**40% smaller L.Occ**	15	12	15	11	○☼	○☼
46	5.8	Craniosynostosis - sagittal	**42% smaller L.Occ**	**35% smaller L.Occ**	17	20	24	16	○☼	○☼
49	5.3	Craniosynostosis - sagittal	**81% smaller L.Occ**	**80% smaller L.Occ**	35	10	32	21	○⊙	○⊙
45	7.4	Craniosynostosis - Crouzons S	**21% smaller L.Occ**	**27% smaller L.Occ**	15	13	14	8	○☼	○☼
44	11.1	Craniosynostosis - multisutural	**72% smaller R.Occ**	**63% smaller R.Occ**	9	9	11	14	☼○	☼○
29	7.2	Craniosynostosis - multisutural	**40% smaller L.Occ**	**44% smaller L.Occ**	12	15	19	16	○☼	○☼
30	4.4	Papilloedema	**68% smaller R.Occ**	**48% smaller R.Occ**	17	27	13	15	☼○	☼○
47	12.9	Papillodema with normal MRI	**23% smaller R.Occ**	18% smaller R.Occ	21	19	24	25	☼○	☼○
38	7.8	IIH	**60% smaller L.Occ**	**95% smaller L.Occ**	17	35	26	43	○☼	○☼
28	8.3	IIH. Optic disc drusen.	**41% smaller L.Occ**	**26% smaller L.Occ**	28	24	25	19	○☼	○☼
33	10.2	Headaches. Optic disc drusen.	**28% smaller R.Occ**	**30% smaller R.Occ**	20	32	18	26	☼○	☼○
21	15.2	Headaches. Blurry vision	**38% smaller L.Occ**	**46% Smaller L.Occ**	8	14	10	10	○☼	○☼
40	8.3	Glioma	8% smaller L.Occ	**57% smaller L.Occ**	14	16	11	11	○☼	○☼
43	8.3	Glioma	**38% smaller L.Occ**	**53% smaller L.Occ**	11	13	14	10	○☼	○☼
26	6.3	Optic neuritis.<br>White matter changes on MRI.	**53% smaller L.Occ**	**43% smaller L.Occ**	25	15	23	17	○☼	○☼
32	10.7	Colour vision changes	**22% smaller R.Occ**	**25% smaller R.Occ**	15	19	15	14	☼○	☼○
22	6	Reduced vision.<br>Pale optic nerves.	**92% smaller L.Occ**	**90% smaller L.Occ**	16	13	10	10	○☼	○☼
37	9.4	Reduced vision.<br>LE central scotoma.	**60% smaller L.Occ**	**65% smaller L.Occ**	14	10	16	9	○☼	○☼
27	11.6	Reduced vision.<br>Subretinal fluid RE.	**21% smaller L.Occ**	17% smaller L.Occ	19	11	18	13	○☼	○☼
35	7.9	Reduced vision.<br>Abnormal foveal reflex.	**23% smaller L.Occ**	**54% smaller L.Occ**	19	10	14	12	○☼	○☼
31	14.6	Reduced vision.<br>Migrane	19% smaller R.Occ	**36% smaller R.Occ**	21	10	20	11	☼○	☼○
25	12.9	Reduced vision.<br>Myopia. Unreliable visual fields.	**20% smaller L.Occ**	**33% smaller L.Occ**	16	17	19	13	○☼	○☼
34	16.9	Reduced vision.<br>Unreliable visual fields	**44% smaller L.Occ**	**45% smaller L.Occ**	5	7	6	8	○☼	○☼
24	8.9	Reduced vision.	19% smaller L.Occ	**56% smaller L.Occ**	19	16	19	18	○☼	○☼
36	9.6	Reduced vision.	16% smaller L.Occ	**53% smaller L.Occ**	12	13	16	15	○☼	○☼
48	11	Reduced vision.	**23% smaller L.Occ**	**43% smaller L.Occ**	10	13	12	11	○☼	○☼
39	10.5	SNHL	**71% smaller R.Occ**	**67% smaller R.Occ**	9	14	12	13	☼○	☼○

TABLE S1. Patient group data. Groups A and B have normal HF-prVEPs. Group C have HF-prVEP defects. The group classification is described in Figure 1, schema of symmetrical (A), asymmetrical (B) FF-prVEPs and HF-prVEP (C).

C.		Abnormal - Half field defect (normal FF-prVEP at Oz and abnormal HF-prVEP) n=11								
ID	Age [years]	Ophthalmology referral	FF-prVEP trans-occipital asymmetry [%]		HF-prVEP iP100 amplitudes [µV]				HF-prVEP distribution	
			RE	LE	RE		LE		RE	LE
					RHF	LHF	RHF	LHF	RHF LHF	RHF LHF
	symbols: ●= hemifield deficit, ○ = normal distribution of hemifields, ⊙ = atypical distribution of hemifields, ☼ = symmetric distribution of hemifields									
13	16.3	Epilepsy pre-surgical evaluation<br>Focal epilepsy	**20% smaller L.Occ**	13% smaller L.occ	5	Absent	5	6	○●	○○
11	14	Glioma	14% smaller R.Occ	**25% smaller L.Occ**	Absent	10	4	Absent	●○	○●
10	13.4	Glioma	0% symmetrical	0% symmetrical	2	9	8	10	●☼	○○
14	9.8	Reduced vision.<br>Craniopharyngioma	12% smaller L.Occ	0% symmetrical	12	14	Absent	17	○○	●☼
9	16.1	Reduced vision.<br>Fibrous dysplasia	18% smaller R.Occ	**33% smaller R.Occ**	5	12	11	10	●○	☼○
8	13.3	Reduced vision. Optic atrophy.<br>Unreliable visual fields.	**36% smaller R.Occ**	**60% smaller R.Occ**	11	12	8	2	☼○	☼●
15	6.9	Reduced vision.<br>Abnormal foveal reflex.	**50% smaller L.Occ**	**70% smaller R.Occ**	15	3	6	18	○●	●○
16	7.6	Reduced vision.<br>Normal MRI.	**31% smaller R.Occ**	9% smaller L.Occ	4	11	12	13	●○	○○
17	5.1	Reduced vision.<br>Bilateral hypermetropia.	**40% smaller L.Occ**	**36% smaller L.Occ**	8	13	11	4	○○	○●
18	5.9	Reduced vision	**64% smaller L.Occ**	17% smaller L.Occ	12	3	10	4	○●	○●
12	8.2	Optic atrophy.<br>RPE changes at the fovea.	**26% smaller L.Occ**	**22% smaller R.Occ**	13	6	4	14	○●	●○
